# Supplementary material for: The Influence of Long-Term Storage on the Epiphytic Microbiome of Postharvest Apples and on Penicillium expansum Occurrence and Patulin Accumulation
Source: Toxins (Basel). 2024 Feb 12;16(2):102. doi: 10.3390/toxins16020102 (PMC10891703; doi:10.3390/toxins16020102)
Supplement: Supplementary file 1 [file toxins-16-00102-s001.zip › toxins-2810669-supplementary.pdf]

Supplementary data

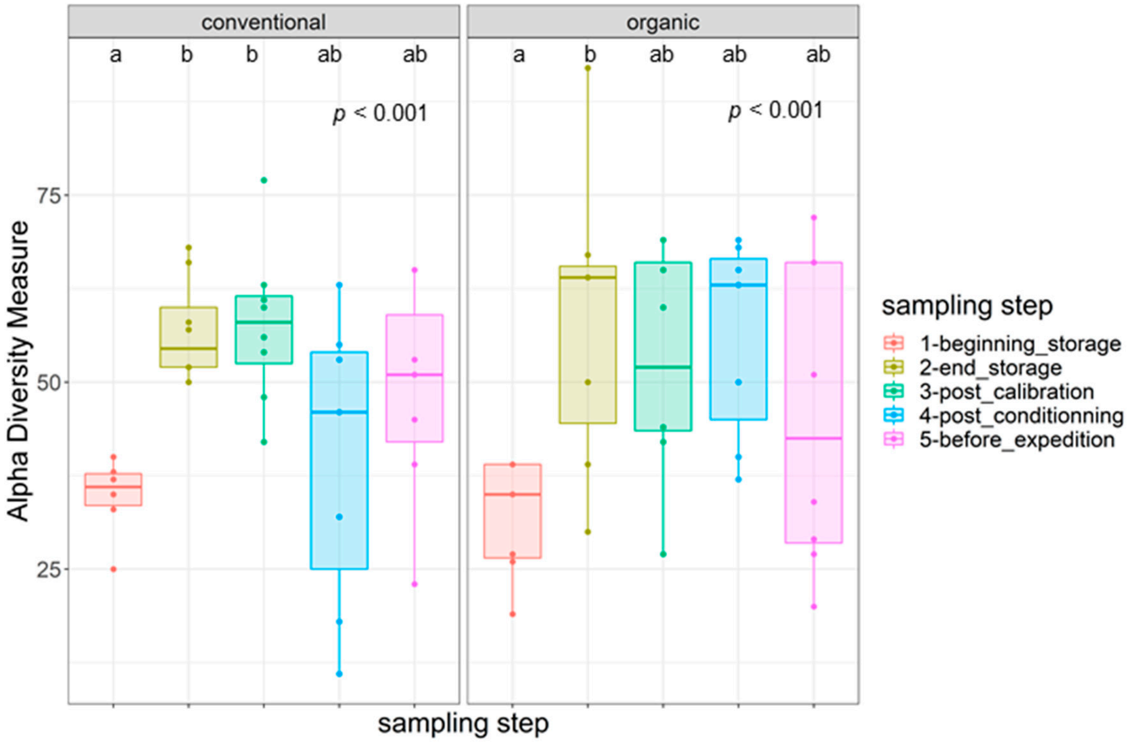

Figure S1. Boxplots illustrating the differences in the Observed alpha diversity measures of the fungal communities on the surface of organically and conventionally grown apples between the different post-harvest stages.

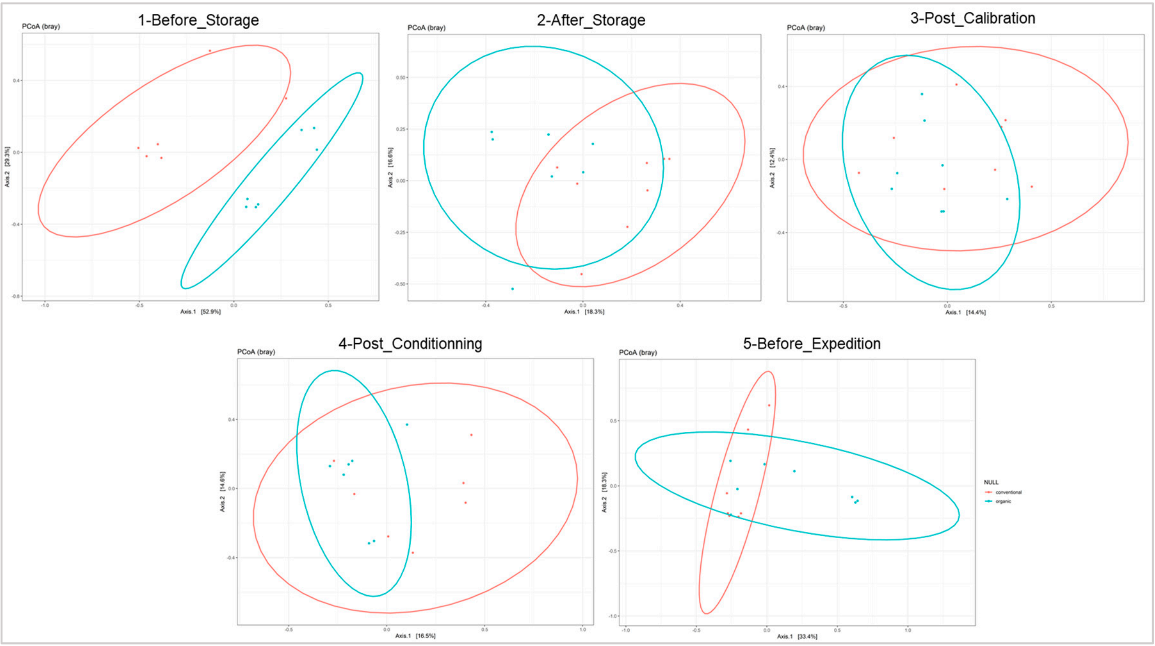

Figure S2. Principal coordinate analysis (PCoA) of fungal populations associated to the surface of conventionally and organically grown apples sampled at the five post-harvest stages based on the beta diversity metric Bray Curtis and divided per sampling step. The PERMANOVA test showed

statistically significant differences in the composition of the fungal communities between the two cultivation systems ($p < 0.001$)

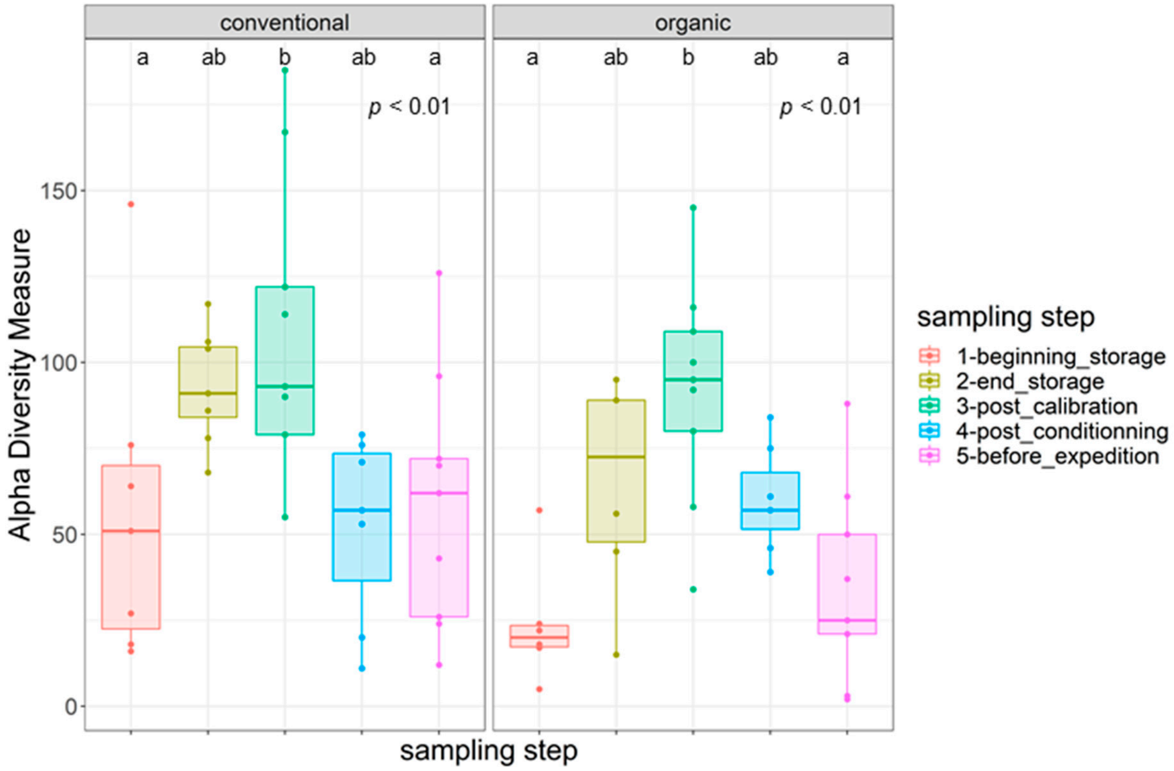

Figure S3. Boxplots illustrating the differences in the Observed alpha diversity measures of the bacterial communities on the surface of organically and conventionally grown apples between the different post-harvest stages.

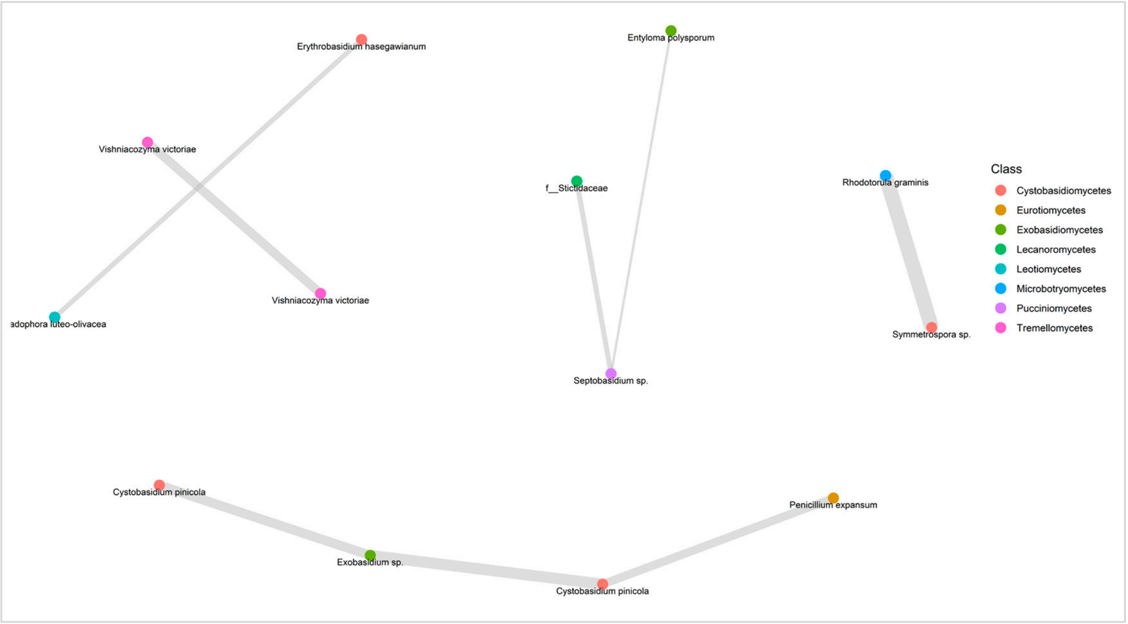

Figure S4. Co-occurrence network diagram (based on SPIEC-EASI) of the fungal species present in at least 5 samples showing competitive associations (negative interactions). Each node represents a different ASV (multiple ASVs could be assigned to the same microbe) and each gray link represents a pairwise co-occurrence. The thickness of the line highlights the degree of interaction between two species, the thickest lines presenting the stronger interactions. When the taxonomic identification

wasn't possible to the species level, the ASV was identified by the lowest possible level of the phylogenetic tree.

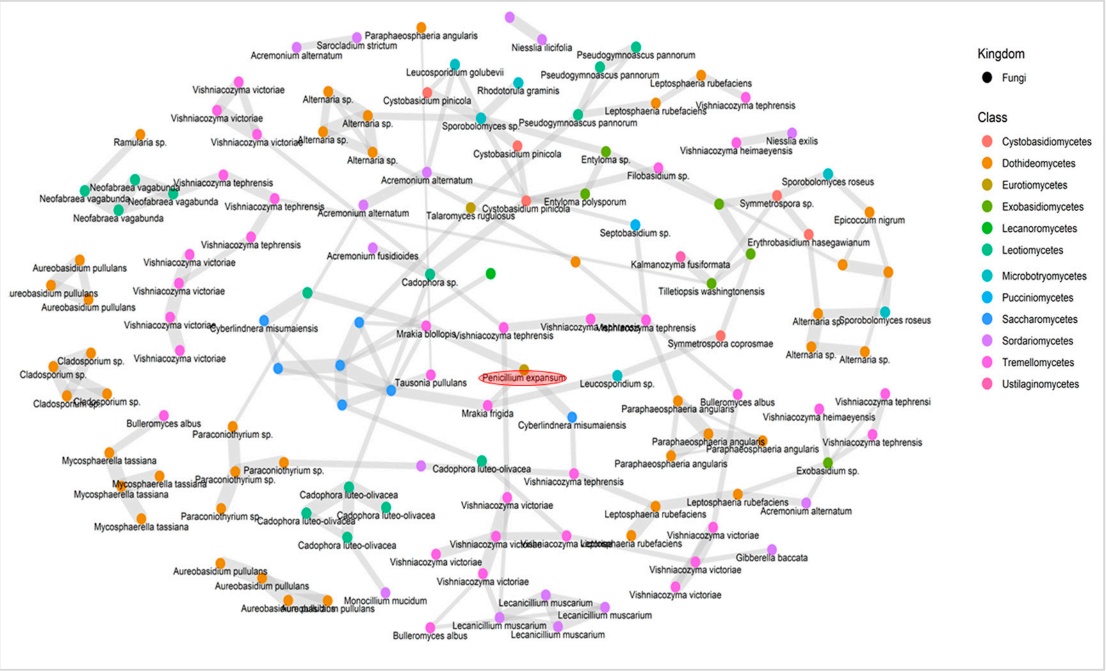

Figure S5. Co-occurrence network diagram (based on SPIEC-EASI) of the fungal species present in at least 5 samples showing cooperative associations (positive interactions). Each node represents a different ASV (two ASVs could be assigned to the same microbe) and each gray link represents a pairwise co-occurrence. The thickness of the line highlights the degree of interaction between two species, the thickest lines presenting the stronger interactions. When the taxonomic identification wasn't possible to the species level, the ASV was identified by the lowest possible level of the phylogenetic tree.
